# Supplementary material for: Functionalized GO Membranes for Efficient Separation of Acid Gases from Natural Gas: A Computational Mechanistic Understanding
Source: Membranes (Basel). 2022 Nov 16;12(11):1155. doi: 10.3390/membranes12111155 (PMC9693057; doi:10.3390/membranes12111155)
Supplement: Supplementary file 1 [file membranes-12-01155-s001.zip › membranes-2010524-supplementary.pdf]

Supporting Information

Functionalized GO membranes for efficient separation of acid gases from natural gas: a computational mechanistic understanding

Quan Liu[1], Zhonglian Yang[1,*], Gongping Liu[2], Longlong Sun[1], Rong Xu[3,*], and Jing Zhong[3]

[1] Analytical and Testing Center, School of Chemical Engineering, Anhui University of Science and Technology, Huainan 232001, China
[2] State Key Laboratory of Materials-Oriented Chemical Engineering, College of Chemical Engineering, Nanjing Tech University, 30 Puzhu Road (S), Nanjing 211816, China
[3] Key Laboratory of Advanced Catalytic Materials and Technology, School of Petrochemical Engineering, Changzhou University, Gehu Road, 213164 Changzhou, China
* Correspondence: zhlyang@aust.edu.cn (Z.Y.); gpliu@njtech.edu.cn (G.L.); xurong@cczu.edu.cn (R.X.)

1. Fitting parameters of unary isotherms with variable adsorption models

Table S1 Fitting parameters of α, γ, β, n and correlation coefficient (R_2) for adsorption isotherms of different gases.

Gases	β	γ	n	R^2
**GO without PDASA**				
N_2	0.001282391	0.000223	1	0.999
CH_4	0.002666389	0.000329		0.999
CO_2	3.390677	1.56608	0.90293	0.994
H_2S	3.859465	1.49488	0.91607	0.995
**GO-1.5wt% PDASA**				
N_2	0.003777	0.00138	1	0.999
CH_4	0.011565	0.00379		0.999
CO_2	4.295758	1.56059	0.90492	0.997
H_2S	4.731904	1.16065	0.96424	0.992
**GO-3.0wt% PDASA**				
N_2	0.003449	0.00126	1	0.999
CH_4	0.010963	0.00343		0.999
CO_2	4.518861	2.49722	0.82063	0.992
H_2S	5.258144	1.72125	0.89405	0.994
**GO-4.5wt% PDASA**				
N_2	0.003589	0.00131	1	0.999
CH_4	0.012041	0.00394		0.999
CO_2	3.694794	2.27132	0.83429	0.989
H_2S	4.789341	1.53676	0.91312	0.996
**GO-6.0wt% PDASA**				
N_2	0.003887	0.00159	1	0.999
CH_4	0.013281	0.00473		0.999
CO_2	3.811163	2.12759	0.85396	0.994
H_2S	4.2226	1.29829	0.94332	0.998
**GO-7.5wt% PDASA**				
N_2	0.003946	0.0016	1	0.999
CH_4	0.013637	0.00492		0.999
CO_2	3.187251	1.37293	0.88646	0.993
H_2S	3.697801	1.07954	0.94362	0.995

2. Logarithmic form of MSD-t curves

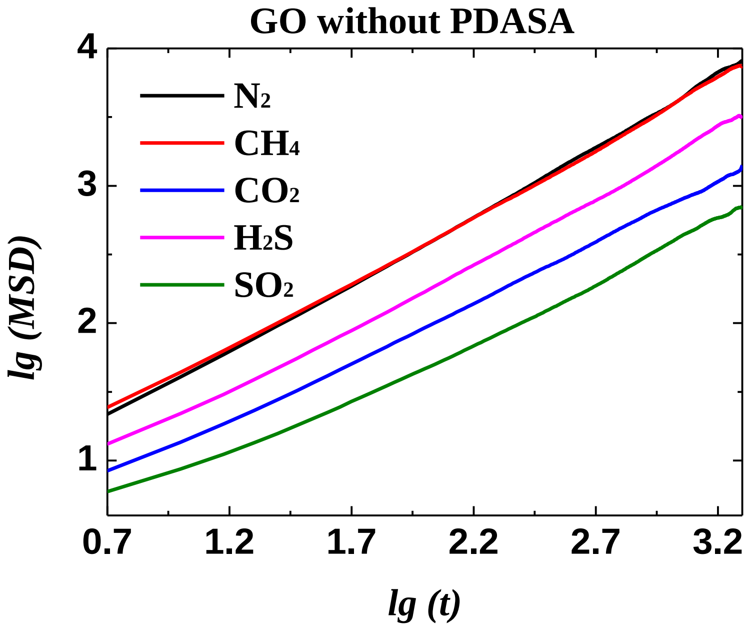

Figure S1. The *lg (MSD)-lg (t)* curve for the transport of variable gases through pure GO and GO-7.5wt%PDASA membranes.

3. Separation performance of different gas through GO membranes

Table S2 The solubility coefficient, diffusion coefficient and permeability with the typically reported unit.

Gases	S ($cm^3(STP)\cdot cm^{-3}\cdot mmHg$)	D (10^{-7} $cm^2\cdot s^{-1}$)	P (Barrer)
**GO without PDASA**			
N_2	0.099598	699.9205	69.710
CH_4	0.299538	662.4247	198.42
CO_2	91.00181	150.2325	13671.42
H_2S	103.5835	210.2658	21780.07
**GO-1.5wt% PDASA**			
N_2	0.102874	553.2705	56.92
CH_4	0.315006	547.9411	172.60
CO_2	117.0023	141.2156	16522.55
H_2S	128.8815	198.2641	25552.57
**GO-3.0wt% PDASA**			
N_2	0.092981	458.2813	42.61
CH_4	0.300666	443.8846	133.46
CO_2	124.8961	93.3594	11660.22
H_2S	145.329	146.3784	21273.03
**GO-4.5wt% PDASA**			
N_2	0.10064	280.9014	28.27
CH_4	0.337283	253.8995	85.64
CO_2	103.5978	70.132	7265.52
H_2S	134.2876	128.0338	17193.36
**GO-6.0wt% PDASA**			
N_2	0.110555	288.3706	31.88
CH_4	0.37771	252.1763	95.25
CO_2	108.3851	37.7374	4090.17
H_2S	120.0859	78.1211	9381.25
**GO-7.5wt% PDASA**			
N_2	0.113789	230.3564	26.21
CH_4	0.393263	219.3084	86.25
CO_2	91.91672	20.6643	1899.40
H_2S	106.6404	16.8647	1798.46

4. Performance comparison

Table S3 Performance comparison for separations of CO_2/CH_4 and $(CO_2+H_2S)/CH_4$.

P_{CO2} (Barrer)	$\alpha_{CO2/CH4}$	$P_{(CO2+H2S)}$ (Barrer)	$\alpha_{(CO2+H2S)/CH4}$	Reference
864	30.7	1180	42	[1]
547	28.2	948	48.9	[2]
599	23.8	1047	42.4	
385	15.2	737	29	[3]
193	18.2	365	34.4	
84.3	24.4	152.3	44	[4]
92	14.9	228	40.9	[5]
76.1	14.2	188.1	35.2	[6]
26	22.3	52.5	45	[7]
142.2	10.35	418	30.43	[8]
100.2	15.24	262.7	39.96	
332	25.7	914.9	40.5	[9]
246.5	26.5	682.4	41.4	
206.9	30	582.2	46.6	
495.1	19	796.1	50.2	
473.5	24.4	671.8	39.7	[10]
496.3	25	823.2	41.5	
543.2	27.1	936.2	46.7	
587.9	29.3	1057.7	52.8	
432.8	26.3	702.3	42.5	
460.4	30.3	747.8	49.2	
460.4	30.3	137.9	52.1	
95.6	36.1	196.1	56.2	
135	38.7	225.9	66.1	
11660.2	87.4	32933.3	246.8	GO-3.0wt% PDASA (This work)
7265.5	84.8	24458.9	285.7	GO-4.5wt% PDASA (This work)

References

1. Qian, Q.; Wright, A.M.; Lee, H.; Dincă, M.; Smith, Z.P. Low-temperature $H_2S/CO_2/CH_4$ separation in mixed-matrix membranes containing MFU-4. *Chem. Mater*. **2021**, *33*, 6825–6831.

2. Liu, G.; Cadiau, A.; Liu, Y.; Adil, K.; Chernikova, V.; Carja, I.-D.; Belmabkhout, Y.; Karunakaran, M.; Shekhah, O.; Zhang, C.; etal. Enabling fluorinated MOF-based membranes for simultaneous removal of H_2S and CO_2 from natural gas. *Angew. Chem. Int. Ed*. **2018**, *57*, 14811–14816.

3. Ahmad, M.Z.; Peters, T.A.; Konnertz, N.M.; Visser, T.; Téllez, C.; Coronas, J.; Fila, V.; de Vos, W.M.; Benes, N.E. High-pressure CO_2/CH_4 separation of Zr-MOFs based mixed matrix membranes. *Sep. Purif. Technol.* **2020**, *230*, 115858.

4. Yahaya, G.O.; Hayek, A.; Alsamah, A.; Shalabi, Y.A.; Ben Sultan, M.M.; Alhajry, R.H. Copolyimide membranes with improved H2S/CH4 selectivity for high-pressure sour mixed-gas separation. *Sep. Purif. Technol.* **2021**, *272*, 118897.

5. Hayek, A.; Alsamah, A.; Alaslai, N.; Maab, H.; Qasem, E.A.; Alhajry, R.H.; Alyami, N.M. Unprecedented Sour Mixed-Gas Permeation Properties of Fluorinated Polyazole-Based Membranes. *ACS Appl. Polym. Mater.* **2020**, *2*, 2199–2210.

6. Hayek, A.; Yahaya, G.O.; Alsamah, A.; Alghannam, A.A.; Jutaily, S.A.; Mokhtari, I. Pure- and sour mixed-gas transport properties of 4,4′-methylenebis(2,6-diethylaniline)-based copolyimide membranes. *Polymer* **2019**, *166*, 184–195.

7. Hayek, A.; Alsamah, A.; Yahaya, G.O.; Qasem, E.A.; Alhajry, R.H. Post-synthetic modification of CARDO-based materials: application in sour natural gas separation. *J. Mater. Chem. A* **2020**, *8*, 23354–23367.

8. Alghannam, A.A.; Yahaya, G.O.; Hayek, A.; Mokhtari, I.; Saleem, Q.; Sewdan, D.A.; Bahamdan, A.A. High pressure pure- and mixed sour gas transport properties of Cardo-type block co-polyimide membranes. *J. Membr. Sci.* **2018**, *553*, 32–42.

9. Liu, Y.; Liu, Z.; Liu, G.; Qiu, W.; Bhuwania, N.; Chinn, D.; Koros, W.J. Surprising plasticization benefits in natural gas upgrading using polyimide membranes. *J. Membr. Sci.* **2020**, *593*, 117430.

10. Liu, G.; Chernikova, V.; Liu, Y.; Zhang, K.; Belmabkhout, Y.; Shekhah, O.; Zhang, C.; Yi, S.; Eddaoudi, M.; Koros, W.J. Mixed matrix formulations with MOF molecular sieving for key energy-intensive separations. *Nat. Mater.* **2018**, *17*, 283–289.
